# Plasma‐Activated Coated Glass: A Novel Platform for Optimal Optical Performance and Cell Culture Substrate Customization

**DOI:** 10.1002/smsc.202300228

**Published:** 2024-01-24

**Authors:** Clara T. H. Tran, Aaron D. Gilmour, Badwi B. Boumelhem, Stuart T. Fraser, Marcela M. M. Bilek

**Affiliations:** ^1^ School of Physics The University of Sydney NSW 2006 Australia; ^2^ School of Biomedical Engineering The University of Sydney NSW 2008 Australia; ^3^ School of Medical Sciences The University of Sydney NSW 2006 Australia; ^4^ Sydney Nano Institute The University of Sydney NSW 2006 Australia

**Keywords:** cell cultures, covalent attachment, glass substrates, plasma‐activated coating, stem cells

## Abstract

Borosilicate glass surpasses polystyrene in optical quality; however, it is less frequently used for cell culture due to poor protein and cell adhesion. To overcome this impasse, the surface of glass coverslips requires functionalization to enable facile covalent attachment of proteins to promote cell attachment and differentiation. Herein, a novel approach is presented to covalently attach proteins to glass by depositing a thin layer of radical‐rich carbon film using a plasma polymerization process. The surface chemistry of these plasma‐activated coatings can be controlled by varying the gas composition used during the deposition. Mass spectrometry reveals different protein profiles attached to functionalized glass coverslips when they are exposed to cell culture media. Mouse embryonic stem cell adhesion and subsequent differentiation into neural lineage on plasma‐treated coverslips are significantly enhanced compared to bare coverslips. Importantly, the coatings are in the nanometer range, preserve the optical properties of the glass coverslips for imaging, and remain stable for at least 4 weeks in simulated body fluid. These results demonstrate the utility of covalently attaching proteins to glass for enhanced cell attachment and stem cell differentiation and provide a promising technique to achieve better outcomes in cell culture in a range of biomedical applications.

## Graphical abstract

Plasma‐activated coatings (PACs) are engineered to apply a radical‐rich carbon film on glass coverslips, allowing covalent protein attachment. Varying the gas composition in the plasma process allows control of diverse protein profiles in cell culture media attached to glass. PAC significantly enhances stem cell adhesion and differentiation while maintaining glass optical properties for optimal optical and fluorescence imaging.

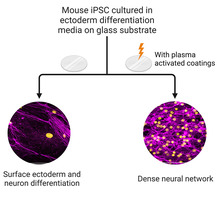

## 1 Introduction

The choice of growth substrates in cell culture is paramount for the success of a cell assay. The specific requirements for cell culture can vary depending on the intended purpose such as cell observation, mass expansion, colony counting, differentiation, gene expression, or drug testing.^[^ ^1^ ^]^ Polystyrene (PS) and glass are the two most extensively used materials for cell culture platforms due to their optical clarity and low autofluorescence which allows for optimal cell observation.

While PS is transparent, it has a slight yellow tint and exhibits autofluorescence in the near UV range.^[^ ^2^ ^]^ On the other hand, borosilicate glass is a highly desirable substrate for high‐resolution and fluorescence imaging, owning to its lower autofluorescence and higher transparency. However, the adhesion of cells to glass is typically weak, hampering the use of glass as a substrate for cell culture. This weak adhesion is primarily attributed to the high hydrophilicity of glass and the lack of diverse functional groups to form bonds with biomolecules. In a study by Milky and co‐workers,^[^ ^3^ ^]^ it was found that 75% of neuronal cells detached from standard glass surfaces after 2 weeks. Many anchorage‐dependent cell lines show slow and highly variable binding to glass coverslips, an ideal substrate for confocal microscopic imaging with short working distance objectives.^[^ ^4^ ^]^ In contrast, cell culture PS plates and dishes with high walls do not fit most confocal imaging systems. Glass may also have profound negative impacts on cells. For example, the growth of human induced pluripotent stem cells (iPSCs) on glass was found with significant levels of chromosomal instability.^[^ ^5^ ^]^ This genetic instability was improved by culturing iPSCs on glass with a nanosheet of PS—a hybrid material combining the advantages of the two materials.^[^ ^5^ ^]^ However, the adoption of this substrate requires time‐consuming microfabrication procedures.

To address this issue, researchers have modified glass surfaces with a layer of extracellular matrix (ECM) proteins such as collagen, laminin, or fibronectin which have been shown to improve cell adhesion and proliferation.^[^ ^6^ ^]^ However, direct physical adsorption of proteins on glass via hydrogen bonds and van der Waals forces presents challenges as it has a high risk of protein detachment during cell culture.^[^ ^7^ ^]^

Covalent attachment of supportive biomolecules to glass is a superior approach for improving the biosignaling and to minimize the loss of these molecules during surface preparation, washing, media changes, and incubation. Various methods have been investigated to improve protein adhesion on glass, including chemical reactions and the addition of functional groups via a plasma polymerization process. The most used method for chemical reaction‐based modification is by aminosilanization using 3‐aminopropyltriethoxysilane (APTES). The process involves soaking the glass in an alkaline solution before silanization with APTES, exposing NH_2_ groups from APTES to adsorb proteins via electrostatic interactions[^6^, ^8^] or covalently attach proteins via chemical linkers.^[^ ^9^ ^]^ However, other polymerization reactions cross‐linking the APTES molecules may occur simultaneously, leaving only a small fraction of NH_2_ groups available for further reactions.^[^ ^10^ ^]^ Plasma polymerization introduces specific functional groups to the surface such as amines, carboxylic acids, aldehydes, and esters through the use of relevant volatile monomers.^[^ ^11^ ^]^ Coupling agents such as carbodiimide and *N*‐hydroxysuccinimide^[^ ^12^ ^]^ react with these functional groups to covalently bind the biomolecules to the surface. Both aminosilanization and functional groups addition through plasma polymerization are time‐consuming processes with multiple steps. While commercially available glass coverslips coated with poly‐d‐lysine, collagen, laminin, gelatin, or fibronectin are an option, they are expensive and lack flexibility in terms of tuning the surface properties required for different cell culture experiments or coating glass surfaces with alternative proteins.

To address these challenges, we have developed new modified glass coverslips which allow users to attach functional biomolecules rapidly and covalently without the need for additional reagents involving toxic linker chemistry. This innovative approach enables researchers to generate surfaces that can be readily optimized for different cell types, providing flexibility and versatility in cell culture experiments. Our method involves the application of a thin and transparent plasma‐activated coating (PAC) onto glass coverslips to serve as an intermediate layer for binding functional biomolecules. Prior studies have demonstrated that PAC can establish covalent bonds with functional biomolecules through reactions with radicals embedded in the coating as a result of energetic ion impacts during the plasma deposition process. The biological functionality of the covalently bound biomolecules is retained due to the mildly hydrophilic nature of the coating. For instance, horseradish peroxidase (HRP) immobilized on a PAC‐treated surface exhibited superior biological functionality compared to that on an untreated surface.^[^ ^13^ ^]^ Similarly, melimine immobilized on PAC‐treated glass demonstrated higher antimicrobial effect compared to melimine adsorbed on untreated glass.^[^ ^14^ ^]^ The covalent attachment of HRP and melimine to PAC was demonstrated by resistance to removal through rigorous stringent washing with sodium dodecyl sulfate at elevated temperatures. In our earlier work, we showed that altering surface charge in different buffer solutions can either increase or reduce the immobilized yeast cell density on plasma‐treated surfaces.^[^ ^15^ ^]^ Additionally, our recent publication highlighted the significant influence of nitrogen content in PAC on DNA and protein attachment density.^[^ ^16^ ^]^ Therefore, modifying the surface chemistry of PAC presents an effective strategy for selectively attaching desired molecules from a mixture, enabling us to tailor the PAC surfaces for various applications.

To customize the surface chemistry of PAC, several parameters can be adjusted during the plasma deposition process, with the gas mixture composition having the greatest influence. In this study, we aimed to characterize PACs deposited from different gas mixtures and investigate their correlation with the differentiation of mouse embryonic stem (ES) cells toward a neural lineage. In vitro cultivation of ES cells requires the replication of key factors to closely mimic the in vivo microenvironment of the target cell lineage. This includes cell signaling events, biochemical elements, and physical cues.^[^ ^17^ ^]^ In conventional assays, cell culture substrates were precoated with ECM proteins such as gelatin,^[^ ^18^ ^]^ followed by ES cell seeding and cultivation in neural media. However, due to the simplicity and efficiency of biomolecule immobilization on PAC in physiological buffers, we condensed the conventional two‐step process of ECM immobilization followed by cell seeding into a single step. In this approach, proteins from the culture media were allowed to adhere to the coverslip surface while the cells settled onto it. Through mass spectrometry analysis of neural media residues on PACs after incubation, we discovered that proteins from neural media adhered differently to PACs with distinct surface chemistries and that these differences had a remarkable impact on the differentiation process.

## 2 Experimental Section

### 2.1 PAC on Glass Coverslips

Glass coverslips (rectangle 22 cm × 22 cm or round 12 mm in diameter, 170 μm thick) were purchased from Livingstone International and sonicated in ethanol before use. The process of surface plasma treatment on glass coverslips comprises two consecutive steps: surface activation and plasma deposition of a thin film coating. To initiate the plasma activation step, we placed coverslips on a stainless‐steel sample holder inside a plasma chamber which was then evacuated to a base pressure of 5 × 10^−5^ Torr. Plasma was generated using a capacitively coupled radio frequency (RF) generator (Eni OEM‐6) with a power of 75 W for 10 min in 75 mTorr of argon. During this step, a pulsed bias of −500 V, with a frequency of 3000 Hz and 20 μs pulse length, was applied to the sample holder using a RUP6 pulse generator (GBS Elektronik GmbH, Dresden, Germany). After the surface activation step, the chamber was again evacuated to the base pressure. The next step involved introducing a mixture of gases (acetylene, nitrogen, and argon) into the chamber. We used four gas mixtures with the same flow rate of acetylene and a constant total flow rate but with varying nitrogen and argon flow rates (see **Table** **1** for details). The pressure during deposition was kept constant at 110 mTorr while the deposition took place for 10 min at 50 W RF power, with the same negative pulsed bias applied by the RUP6 power supply. After the plasma deposition, samples were stored in a petri dish under ambient laboratory conditions for further analysis.

**Table 1:** Four recipes with different gas mixtures used in the plasma deposition process

Recipe	Acetylene [sccm]	Nitrogen [sccm]	Argon [sccm]
No_N	1	0	13
Low_N	1	3	10
Mid_N	1	10	3
High_N	1	13	0

### 2.2 Surface Characterization of PAC

Contact angles of two liquid probes (water and diiodomethane) were measured on the plasma‐coated (denoted as PAC) and ethanol‐cleaned glass coverslips and PS film (0.19 mm, Goodfellow Ltd., Cambridge) using a Theta tensiometer (ATA Scientific Instruments). The average of five contact angles of each liquid probe was used to calculate surface energy using the Owens–Wendt–Rabel–Kaeble method. PAC samples were measured 1 day and 3 months after deposition, during which the samples were stored in ambient laboratory conditions.

Surface chemistry of the coatings was investigated using transmission Fourier transform infrared spectroscopy (T‐FTIR) and X‐ray photoelectron spectroscopy (XPS). T‐FTIR was used to obtain spectra of PAC deposited on high purity silicon wafers (B‐doped p‐type, 1–10 Ω cm resistivity, 100 orientation) 1 day after deposition. High purity silicon wafers were chosen for the T‐FTIR measurements because they have similar surface chemistry to glass with low infrared absorbance. FTIR spectra were collected with 500 scans at a resolution of 4 cm^−1^ in vacuum using a Vertex 70v spectrometer (Bruker). Spectra of PAC were obtained by subtracting the spectrum of the bare silicon wafer from the spectra of PAC on the silicon wafer. For XPS analysis, PAC was deposited on thin oxide silicon wafers and analyzed after 1 week in laboratory ambient. Thin oxide silicon wafer was chosen as a substrate for this analysis to reduce surface charge accumulation during the measurement. Survey scans (step size of 1 eV) and high‐resolution scans of C 1*s*, N 1*s*, O 1*s*, and Si 2*p* (step size of 0.1 eV) were recorded using a Thermo Scientific K‐Alpha+ spectrometer (Thermo‐Fisher Scientific, UK). Areas of the peaks from high‐resolution scans were integrated to calculate the atomic percentage of elements on each surface. The C 1*s* peaks were fitted with Lorentzian–Gaussian peaks using the following criteria: all deconvoluted peaks have the same full width half maximum and same ratio of Lorentzian/Gaussian mix, binding energy of C─C, C─O, C═O, and O─C═O are approximately 1.5 eV apart, respectively.

The optical properties of PAC were characterized using spectroscopic ellipsometry and transmission spectrophotometry. PAC‐coated silicon wafers (B‐doped p‐type, 1–100 Ω cm resistivity, 100 orientation) were used for ellipsometry measurement (J.A. Woollam M2000) with three angles (65°, 70°, and 75°). Psi and delta plots were fitted using a Cauchy layer to obtain the coating thickness and refractive index of the coatings. To study the transparency of PAC, we scanned PAC‐coated glass coverslips in the visible and UV range using a Cary spectrophotometer (Agilent Technologies) with an ethanol‐cleaned glass coverslip used as a control. To evaluate the autofluorescence of the coatings, PAC‐treated and bare coverslips were placed facing up in a 24 well plate and the level of autofluorescence was measured using a CLARIOstar (BMG Labtech) plate. Preset excitation and emission bands were selected for five common fluorophores (see **Table** **2**) while gain values were manually set. Fluorescence emission was recorded from 32 spots at a radius of 6 mm from the center of the well.

**Table 2:** Excitation and emission wavelengths of the five fluorophores used for autofluorescence measurements

Preset name	Excitation [nm]	Dichroic filter [nm]	Emission [nm]	Gain
DAPI/DNA	360/20	auto 407.5	460/30	500
Alexa Fluor 488	488/14	auto 507.5	535/30	1000
Alexa Fluor 555	535/20	auto 557.5	585/30	1000
Alexa Fluor 633	605/30	auto 632.5	670/50	1000
Alexa Fluor 647	625/30	auto 652.5	680/30	1000

### 2.3 Radical Decay of PAC During Storage and Ability to Form Covalent Bonds with ECM Proteins

Concentration and nature of unpaired electrons (radicals) in PAC coatings were investigated using electron spin resonance (EPR) (SpinScanX, Adani, Belarus). PAC was deposited on quartz slides (40 × 50 × 0.9 mm) which were fitted into the resonance cavity of the EPR equipment. We measured samples at different time points during storage to investigate radical decay over time. The measurements were conducted at room temperature with a microwave frequency of 9.40 MHz and a central magnetic field of 336 mT with a sweep width of 30 mT and a microwave power of 0.946 mW. The peak areas which correlate to radical concentration were integrated using Origin Pro software and the *g*‐factor was calculated using microwave frequency and peak position. The reported results are an average of three measurements at each time point.

To demonstrate the covalent attachment of an ECM at desired locations on PAC, we used microcontact printing of gelatin and a stringent wash with detergent to test the bond strength. Polydimethylsiloxane (PDMS) stamps were prepared by lithography with parallel stripes of 50 μm width with 100 μm gaps in between. They were treated with oxygen plasma (Harrick Plasma) for 3 min before use. Fluorescein‐conjugated gelatin from pig skin (Life Technology Australia) was prepared at a concentration of 0.1 mg mL^−1^ in phosphate buffer saline (PBS) buffer. A drop of 20 μL gelatin solution was placed on a PDMS stamp and incubated at room temperature. After 30 min, stamps were briefly rinsed with PBS and the excess liquid on the stamp surface was absorbed using Kim wipes. The stamps were placed onto the sample surfaces (UT and PAC) with a small weight support (≈30 g) to enhance the contact for 10 min. Samples were subsequently rinsed with milli‐Q water on a shaker (30 rpm) for 30 min to remove unbound molecules before drying for confocal fluorescence microscopy. A more stringent wash with 5% sodium dodecyl sulfate (SDS) was used to remove physisorbed molecules on the sample surface and to identify covalently bound molecules.

Confocal images were collected using Leica SP5 (Leica) microscope. Images were acquired using a 20× water immersion objective at either 1× magnification (low resolution) or 4× magnification (high resolution) with a 488 nm excitation laser.

### 2.4 Stability of the PAC on Glass

The stability of PAC adhesion on glass is important for successful cell culture, which typically occurs in culture media over several weeks. To assess the stability of the coating in simulated body fluid (SBF) for a month, we conducted a test by comparing the surface composition of samples before and after a 4 week soak in SBF. We utilized glass slide cuttings (2.5 cm × 1.5 cm) as substrates for plasma coatings with four PAC recipes, as described in Section 2.1. The samples were stored in a 6‐well plate at ambient conditions for 1 week before the experiment. To prevent contamination, we prepared and filtered the SBF using a 0.22 μm microfilter, and treated the samples with UV light (10 min each side) in a safety cabinet before adding SBF (4 mL well^−1^). The well plate was then wrapped in parafilm and placed in a 37 °C incubator for 4 weeks with SBF replaced weekly to maintain its concentration. After the incubation, samples were dried and analyzed by XPS at three different locations to ensure the consistency of the measurement. Statistical analysis was performed using unpaired *t*‐test from GraphPad Prism. The presence of the coating was indicated by the presence of nitrogen and carbon, which are elements in PAC. The reduction in amount of nitrogen and carbon and the increase of silicon after incubation would indicate coating loss, otherwise the coating is still intact.

### 2.5 Attachment and Differentiation of Mouse ES Cells on PAC Glass Coverslips

Wild‐type, undifferentiated mouse ES cells (129/SvJ)^[^ ^19^ ^]^ were maintained in mouse ES cell maintenance media including Dulbecco's Modified Eagle Medium (DMEM) supplemented with 10% v/v fetal calf serum (Bovogen), 2 mm Glutamax (ThermoFisher Scientific), 1 mm sodium pyruvate (ThermoFisher Scientific), 50 000 U mL^−1^ penicillin, 50 mg mL^−1^ streptomycin (ThermoFisher Scientific), and 150 μm of 1‐thioglycerol (Sigma–Aldrich). Leukemia inhibitory factor (ProspecBio) was added at a concentration of 100 units mL^−1^ to inhibit spontaneous mouse ES cell differentiation.

The mouse ES cell maintenance media was removed by aspiration, and the cells were washed with 1 mL PBS. After that, 500 μL of TrypLE Express was added to replace PBS and cells were incubated at 37 °C for 3 min to initiate dissociation. To stop the dissociation process, 1.5 mL of mouse ES cell maintenance media was added. The cell mixture was transferred into a 15 mL Corning tube and centrifuged at 240 × g for 5 min. The supernatant was discarded, and the cell pellet was resuspended in 1 mL of maintenance media.

To induce differentiation of mouse ES cells toward a neural lineage, 2.5 × 10^4^ cells were plated on PAC glass coverslips distributed across the four different recipes outlined above as well as a control (untreated glass coverslips). Mouse ES cells were supplemented with neural induction media.^[^ ^20^ ^]^ Briefly, media consisted of 50:50 DMEM/F12: neurobasal (ThermoFisher Scientific) media supplemented with 1× N2 (ThermoFisher Scientific), 1× B27 (ThermoFisher Scientific), 1 mM Glutamax, 50 000 U mL^−1^ penicillin, 50 mg mL^−1^ streptomycin, 2.5 mm sodium ascorbate (Sigma–Aldrich), and 450 μm of 1‐thioglycerol. Media was changed every 2 days to promote cell survival until day 22 of differentiation. The total numbers of neuronal cell clusters, neurites, and neurite length were measured using ImageJ and individual values were plotted using GraphPad Prism (*n* = 4, three independent experiments). Data comparing the size and number of neurites differentiated from mouse ES cells are presented as mean ± SEM. GraphPad Prism 7 was used to generate graphs and determine statistical significance. A two‐tailed one‐way ANOVA with Tukey's post hoc analysis was used to compare the size and number of neurites differentiated between the untreated coverslips and PAC glass coverslip formulations. A *p*‐value less than 0.05 was deemed significant.

After 22 days, media was aspirated and differentiated mouse ES cells were washed twice with PBS. Following, differentiated cells were fixed with 4% paraformaldehyde (in PBS) for 10–20 min at room temperature. Coverslips were then washed 3 times with PBS and once with PBS supplemented with 1% v/v Triton‐X (PBST). Differentiated cells were then stained with 1 μm of Alexa Fluor^TM^ 555 Phalloidin (Sigma–Aldrich) in PBST overnight at 4 °C. The following day, Phalloidin‐stained cells were washed 3 times with PBS and mounted in Vectashield containing 4′,6‐diamidino‐2‐phenylindole (DAPI) (Vector Laboratories, USA). High‐resolution images were taken using a Zeiss LSM 800 confocal microscope coupled with the Zen Blue software package. Phalloidin was excited using the 565 nm laser while DAPI was excited using the 405 nm laser. Images were taken at 20× magnification. A 3 × 3 area focused on the center of the coverslip was imaged and stitched together. Postprocessing of images was performed on ImageJ (NIH) and data was graphed using GraphPad Prism 7.

### 2.6 Mass Spectrometry Analysis

PAC and UT glass coverslips were incubated in complete neural induction media (Section 2.4 above) for 1 h at 37 °C in a cell culture incubator. Complete media was removed and the coverslips were washed in either PBS or 5% SDS for 1 h at 37 °C on an orbital shaker and then rinsed with PBS before being transferred to a clean 96 well plate and stored at −20 °C. These coverslips were later sent to Sydney Mass Spectrometry for 1D liquid chromatography mass spectrometry (LC/MS).

Coverslips were stored overnight at 4 °C prior to digestion in 12 ng μL^−1^ trypsin and 25 mm ammonium bicarbonate at 37 °C overnight. Samples were ZipTipped and eluted in 50% acetonitrile and 0.1% trifluoroacetic acid. The eluate was dried down and reconstituted in 5 μL loading buffer. Samples were subsequently diluted 1:10 and 3 μL was loaded onto LC/MS (QSTAR, Applied Biosystems). Acquired spectra were processed using the MASCOT databases for human and bovine. Identified proteins with low peptide spectrum match (PSM) counts found on only one sample were excluded from further analysis. Additionally, human keratin hits were also excluded, as it is an environmental contaminant.

## 3 Results

### 3.1 Optical and Chemical Properties of PAC‐Treated Glass Coverslips

The transparency of glass coverslips plays a crucial role in the quality of microscopy images. The thickness of the PAC varied according to the gas mixture composition used during the deposition process. PACs deposited with No_N, Low_N, and Mid_N coatings had thicknesses ranging between 7 and 15 nm while the High_N coating had a thickness range of 20–40 nm. Notably, these thicknesses are significantly thinner than the glass coverslip itself.

**Figure 1:**
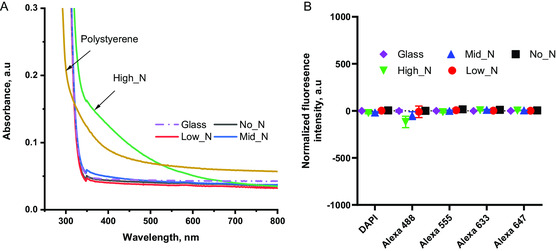
A) Optical absorbance of PACs in the UV and visible wavelength range in comparison with a cleaned glass coverslip (denoted as glass) and polystyrene (denoted as PS). All PAC‐coated glass coverslips have similar absorbance to glass except the one with High_N coating. B) Normalized autofluorescence of PACs relative to glass for a range of standard fluorescence filter sets. The autofluorescence of all PAC recipes were equivalent to the background autofluorescence of glass coverslips (fluorescence detector dynamic range: 0–260 000).

The absorbance of the different PAC on glass coverslips in the UV and visible wavelength ranges was compared with those of a cleaned glass coverslip and PS film (0.19 mm) (**Figure** **1**A). All PACs had similar absorbance values to the cleaned glass coverslip and had lower absorbance values than PS except the High_N PAC glass coverslip which had a higher absorbance in the 320–600 nm range. Autofluorescence of PACs on glass was investigated at five different excitation/emission ranges of commonly used fluorophores. Figure 1B shows the fluorescence intensities of PACs relative to glass which vary in very narrow ranges compared to the fluorescence dynamic range of the detector. Our finding suggests that the use of PAC on glass has a negligible effect on the transparency and autofluorescence of glass substrate and therefore is suitable for high‐quality imaging, including when using fluorescence staining.

**Figure 2:**
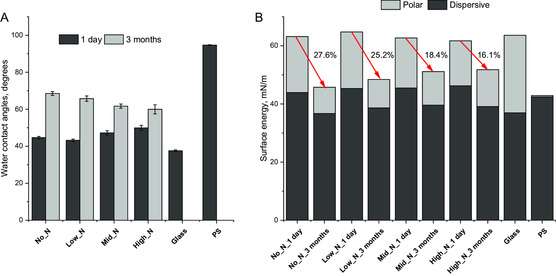
Comparison of A) water contact angles and B) surface energies of PACs including their dispersive and polar components calculated using the Owens–Wendt–Rabel–Kaeble method. Glass and PS were used as controls and do not show significant variation over storage time. Water contact angles of PACs vary between 40 and 50 °C just after the plasma deposition and progress to 60–70 °C after 3 months of storage. Surface energy of PAC reduces during storage with the largest reduction from no nitrogen containing gas composition.

The conformation of immobilized proteins can be greatly influenced by the wettability of the substrates in which mildly hydrophilicity (approximately 60°) is preferred for protein attachment. Our measurement shows that all PAC‐coated glass coverslips have water contact angles ranging between 40° and 50° (**Figure** **2**A) which are slightly higher than cleaned glass but significantly lower than PS (94°). Among the PACs, the High_N recipe has the highest contact angle. Total surface energies of PAC‐treated glass samples are similar to clean glass; however, the polar energy is lower while the dispersive component of the surface energy is higher (Figure 2B). After 3 months of storage under ambient conditions, the water contact angles on all PAC coatings increased to 60–68° while the total surface energy decreased (Figure 2), with its polar component reducing more than the dispersive component.

The total surface energy of a surface is indicative of the attractive forces governing molecule interactions. The dispersive component arises from the temporary fluctuations in the electron cloud distribution around the molecules, resulting in induced dipoles that attract neighboring molecules. In contrast, the polar component is rooted in the attraction of permanent dipoles. The polar surface energy of PACs comprises contributions from polar groups present on the surface and unpaired electrons.^[^ ^21^ ^]^ Polar groups, especially oxygen containing groups, were identified through the use of FTIR spectroscopy while unpaired electrons were detected using EPR spectroscopy, as will be shown subsequently. The observed reduction in the polar component appears to be directly associated with a decrease in detected radicals within PACs during storage and it is correlated with nitrogen gas ratio used in the plasma deposition process. These findings suggest that PAC‐treated glass coverslips remain hydrophilic after the treatment and that the nitrogen ratio in the gas composition used during plasma deposition may play an important role in determining the long‐term stability of PAC.

**Figure 3:**
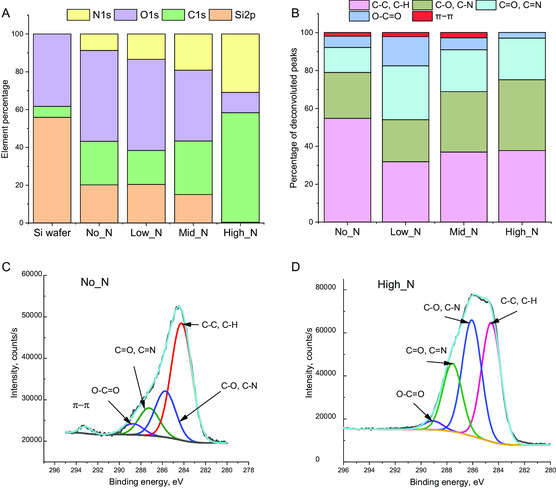
XPS analysis of PACs on silicon wafers. A) Atomic percentages of elements detected on the surfaces show the increasing nitrogen contents when more nitrogen was used in the plasma deposition process. B) Percentage of deconvoluted peaks from C 1*s* high‐resolution peaks. Low_N shows the highest content of COO‐ group. C) Deconvolution of C 1*s* obtained from No_N and D) deconvolution of C 1*s* obtained from High_N coatings.

XPS analysis was conducted to compare the elemental composition of the coatings deposited on silicon wafers (**Figure** **3**). A small amount of carbon was detected on bare silicon wafers, likely from a thin layer of adventitious carbon from environmental contamination. Silicon wafers typically have a native oxide layer (approximately 2 nm thick) where oxygen was detected. XPS analysis on the PAC‐coated, silicon wafers showed a small signal of silicon on all samples except for the High_N sample. This is because the High_N coating is thicker than the other coatings and XPS is most sensitive to approximately the top 10 nm of the sample. On samples where silicon was detected, the oxygen could have originated from the PAC layer and the SiO_2_ layer underneath. Nitrogen is the only element that is contained in the plasma coating. Figure 3A shows that nitrogen content (yellow bars) increases from left to right, corresponding to the increasing levels of nitrogen used in the gas mixtures. The highest nitrogen level is 30% obtained from deposition in a gas mixture of acetylene and nitrogen only. This coating also has the highest carbon content and lowest oxygen content. We further deconvoluted C 1*s* peaks to obtain carbon bond types. Figure 3C,D show examples of C 1*s* peaks from the No_N and High_N samples respectively. All C 1*s* peaks contain four or five deconvoluted peaks correlated to carbon bonding to carbon, oxygen, and nitrogen (Figure 3B). Among these, Low_N has the highest percentage of O*─*C═O groups while High_N has the lowest. Surfaces with O*─*C═O groups tend to have a more negative charge in buffer solutions while those with more nitrogen tend to have a more positive charge in an appropriate buffer.

**Figure 4:**
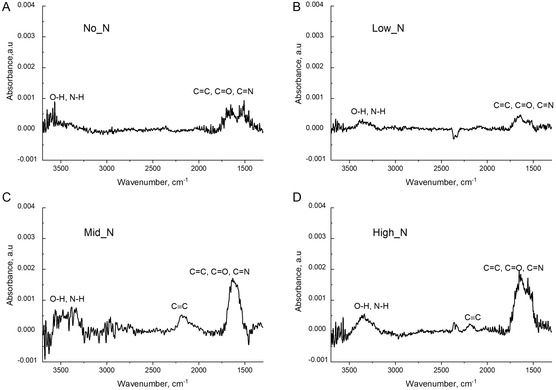
FTIR spectra of PAC produced with different gas mixture compositions showing the identification of functional groups from the absorbances in the mid‐infrared range. A) No_N; B) Low_N; C) Mid_N and D) High_N.

The FTIR analysis (**Figure** **4**) further shows absorbances in the range of 3600–3250 cm^−1^ which can be attributed to the vibrations of hydrogen bonds from O*─*H and N*─*H groups. The absorbance observed in the 1750–1500 cm^−1^ range correlates to unsaturated carbonyl groups including C═O, C═N, and C═C. Notably, there was a minor absorbance at 2200 cm^−1^ which is associated with C≡C bonds. This triple bond is not observed in No_N and Low_N coatings, suggesting that a gas mixture with high argon can break down acetylene more effectively than a gas mixture with low or no argon.

### 3.2 Radical Density in PACs

**Figure 5:**
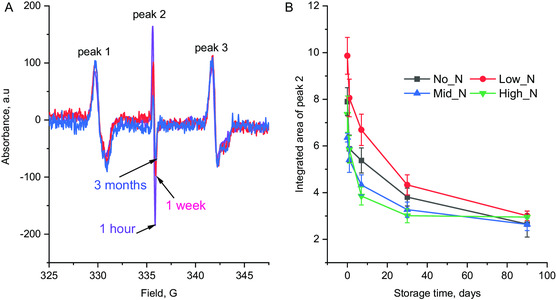
Comparison of electron spin resonance measurements. A) First derivative of EPR signal of Low_N coating just after the treatment, 1 week and 3 months, showing radical decay of peak 2 over time. B) Comparison of peak 2 area during storage time measured on four PACs. The radicals in each PAC decay with different rate but reach the same level after 3 months.

The density of radicals plays a critical role in determining the covalent attachment of biomolecules on the PACs. EPR measurement on quartz slides coated with PAC showed the presence of three absorption peaks (**Figure** **5**A); among those, only peak 2 displayed a change over the storage time while peaks 1 and 3 remained constant. As our plasma treatment includes two steps, surface activation using argon plasma and plasma deposition using a gas mixture, we measured EPR on quartz after the first step to identify the effect of argon plasma. We found peaks 1 and 3 appeared with the same intensity as those detected on PAC‐coated quartz, whereas peak 2 was negligible. Based on this observation, we assume that peaks 1 and 3 correspond to radicals generated in the substrate (quartz) as the result of argon ion bombardment during the first step (surface activation process), while peak 2 mainly originates from the plasma deposition process.

Figure 5B depicts the decay of radicals over time under ambient conditions, measured by the reduction in the integrated area of peak 2. This peak decreased sharply within the first day and continued to decline over the course of a month before reaching a slower rate of decay. PAC coated with the Low_N gas mixture exhibited the highest radical content during the first month of storage, whereas Mid_N and High_N samples showed the lowest radical content. Despite the initial radical content variation, all PACs exhibited a similar level of radicals after 3 months of storage. This suggests that gas mixtures with high argon ratios (No_N and Low_N) produce thinner coatings but with higher number of radicals compared to those with high nitrogen ratios. This trend can be attributed to the larger size and inertness of argon, which results in a greater momentum transfer on impact during ion bombardment, generating more broken bonds whilst not eliminating radicals by bonding into the surface structure as the nitrogen atoms do.

### 3.3 Stability of PAC on Glass in SBF

**Figure 6:**
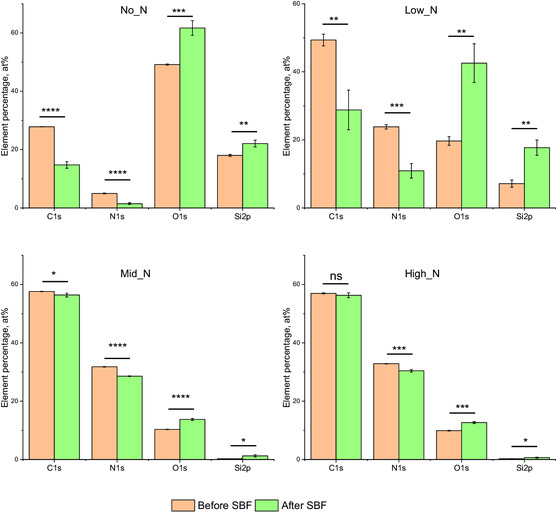
Comparison of plasma coating components from XPS analysis of samples before and after soaking in SBF at 37 °C for 4 weeks. High nitrogen content coatings are more stable in SBF. Data are the average of three random spots on the coatings. *p < 0.05; **p < 0.01; ***p < 0.001; ****p < 0.0001; ns, *p* > 0.05 (nonsignificant).

Stability of the plasma coatings in SBF at 37 °C was evaluated from the surface chemical components analyzed before and after 4 weeks in this solution (**Figure** **6**). Significant changes of element percentage were observed on No_N and Low_N coatings, whereas negligible changes were found on Mid_N and High_N coatings. In comparison to bare glass, which has 9.58 ± 0.72% carbon, 0.33 ± 0.02% nitrogen, 62.34 ± 0.43% oxygen, and 27.74 ± 0.30% silicon, the No_N and Low_N coatings still exhibit higher carbon and nitrogen contents, indicating that the coatings were still present on the glass surface but thinner. These data suggest that coatings with high nitrogen content are more stable in SBF.

### 3.4 Nature of Protein Attachment on PAC in Comparison with Bare Glass

**Figure 7:**
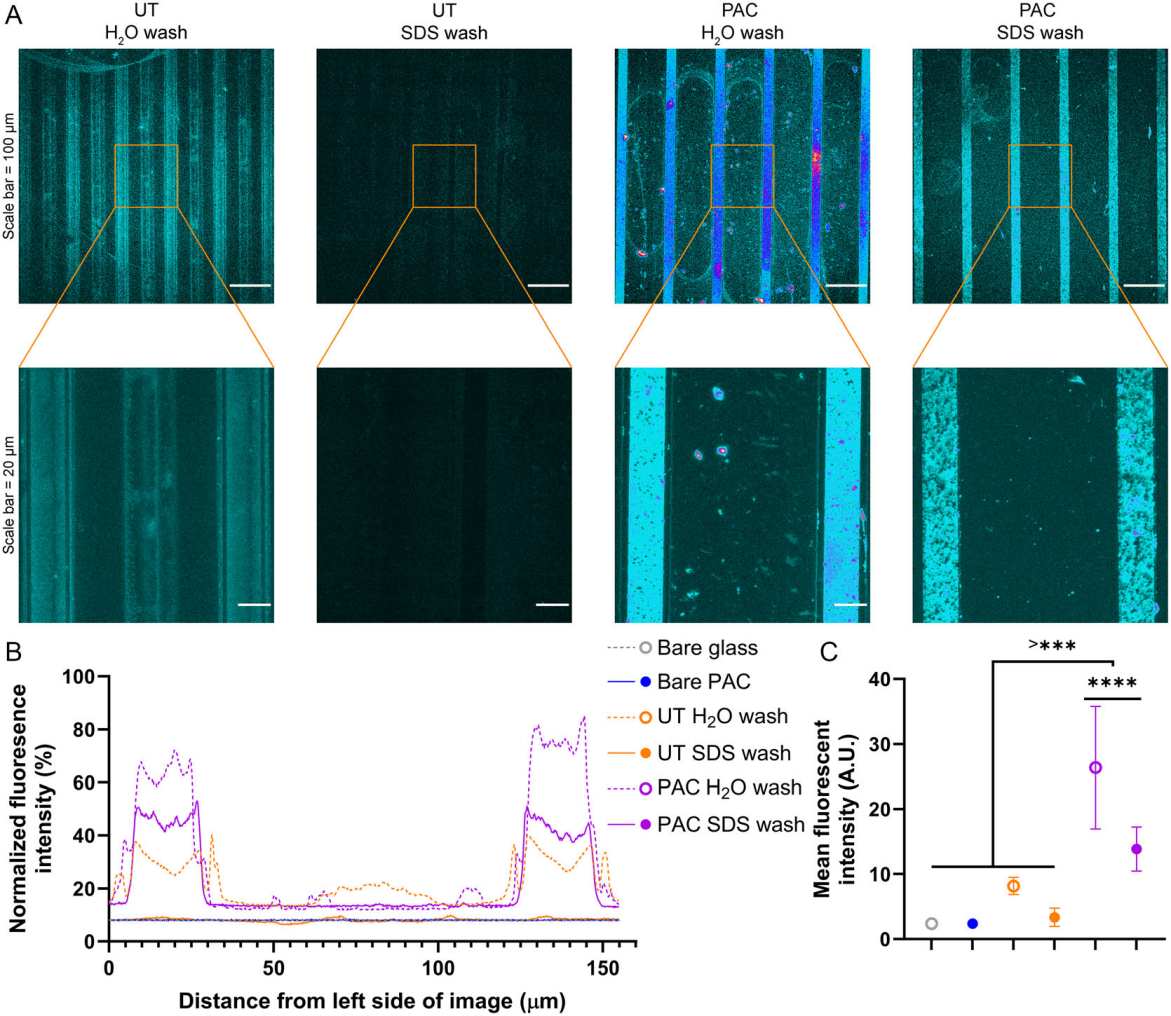
Comparison of microcontact printing of fluorescently labeled gelatin on bare glass and PAC using confocal microscopy. A) Comparative fluorescent micrographs at low resolution and high resolution. Images are color coded based on fluorescence intensity, black‐cyan = low intensity, pink‐yellow = high intensity. B) Mean fluorescent intensity from high‐resolution images, *x*‐axis indicates distance from the left edge of each image. C) Statistical analysis of mean fluorescent intensity from high‐resolution images: ***p < 0.001; ****p < 0.0001.

Microcontact printing of fluorescently labeled gelatin on the PAC and cleaned glass (denoted as UT) were compared after mild washing with water or stringent washing with SDS (**Figure** **7**). Gelatin on cleaned glass after washing with water showed poor edge fidelity as evidenced by the prominent shoulder regions flanking either side of the main peaks in Figure 7B (green line). After washing with SDS the fluorescent signal was close to control samples without gelatin. On the contrary, PAC samples demonstrated much improved edge fidelity and after SDS washing showed a decrease in fluorescent intensity, but still exhibited sharp and bright printed lines (Figure 7A), indicating covalent binding of gelatin to the surface.

### 3.5 PAC Treatment of Coverslips Efficiently Promotes Neural Differentiation of Mouse ES Cells

**Figure 8:**
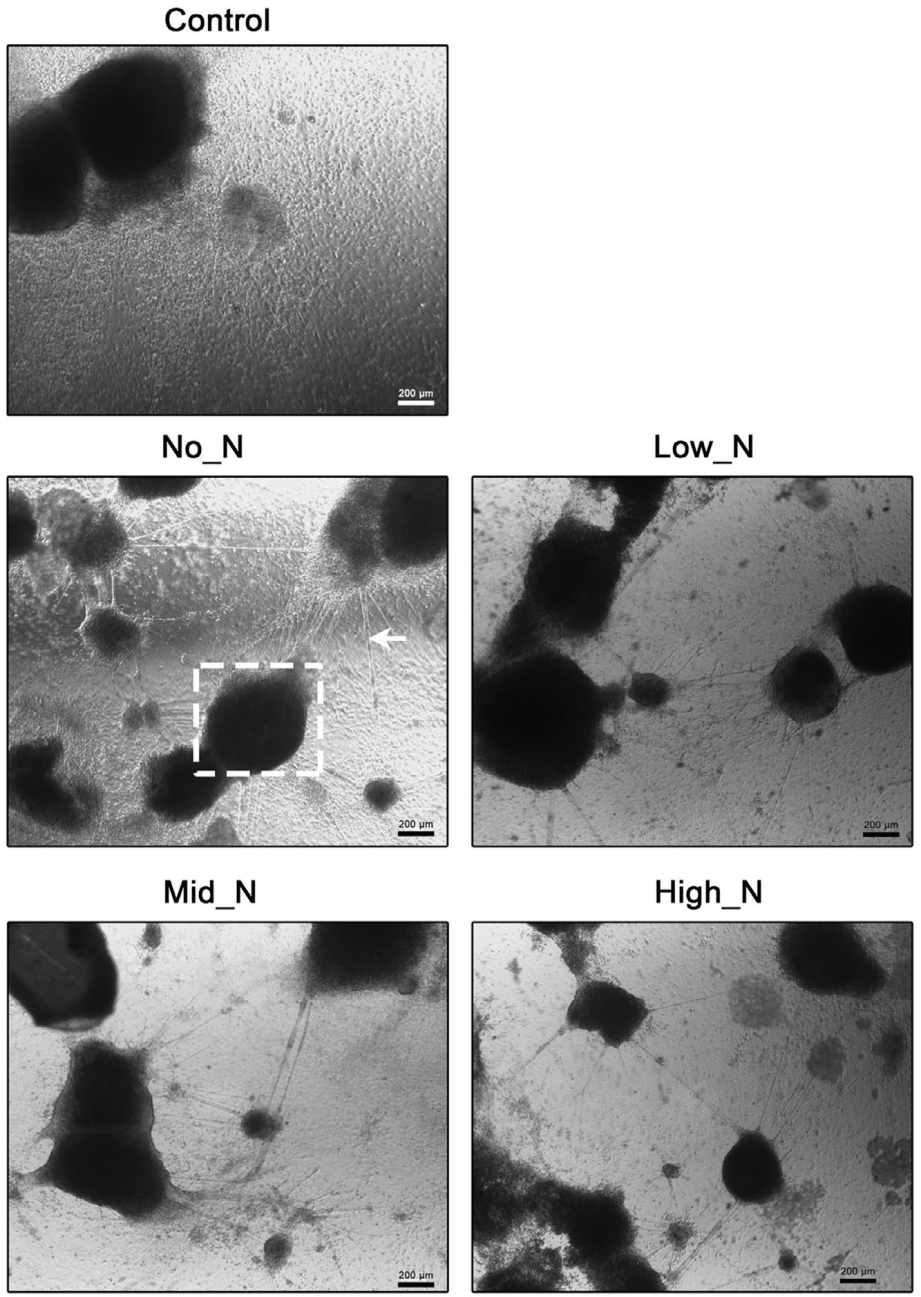
Large embryoid bodies with neurite‐like projections differentiate from mouse ES cells grown on PAC glass. Mouse ES cells were plated on bare glass (control) and different formulations of PAC for 22 days. Representative phase contrast images were taken at 10x magnification on a Zeiss Axiovert microscope coupled with the Zen Blue software package. Dotted box denotes a neuronal cluster while the arrowhead denotes neuronal projections. Scale bars indicate a length of 200 μm.

**Figure 9:**
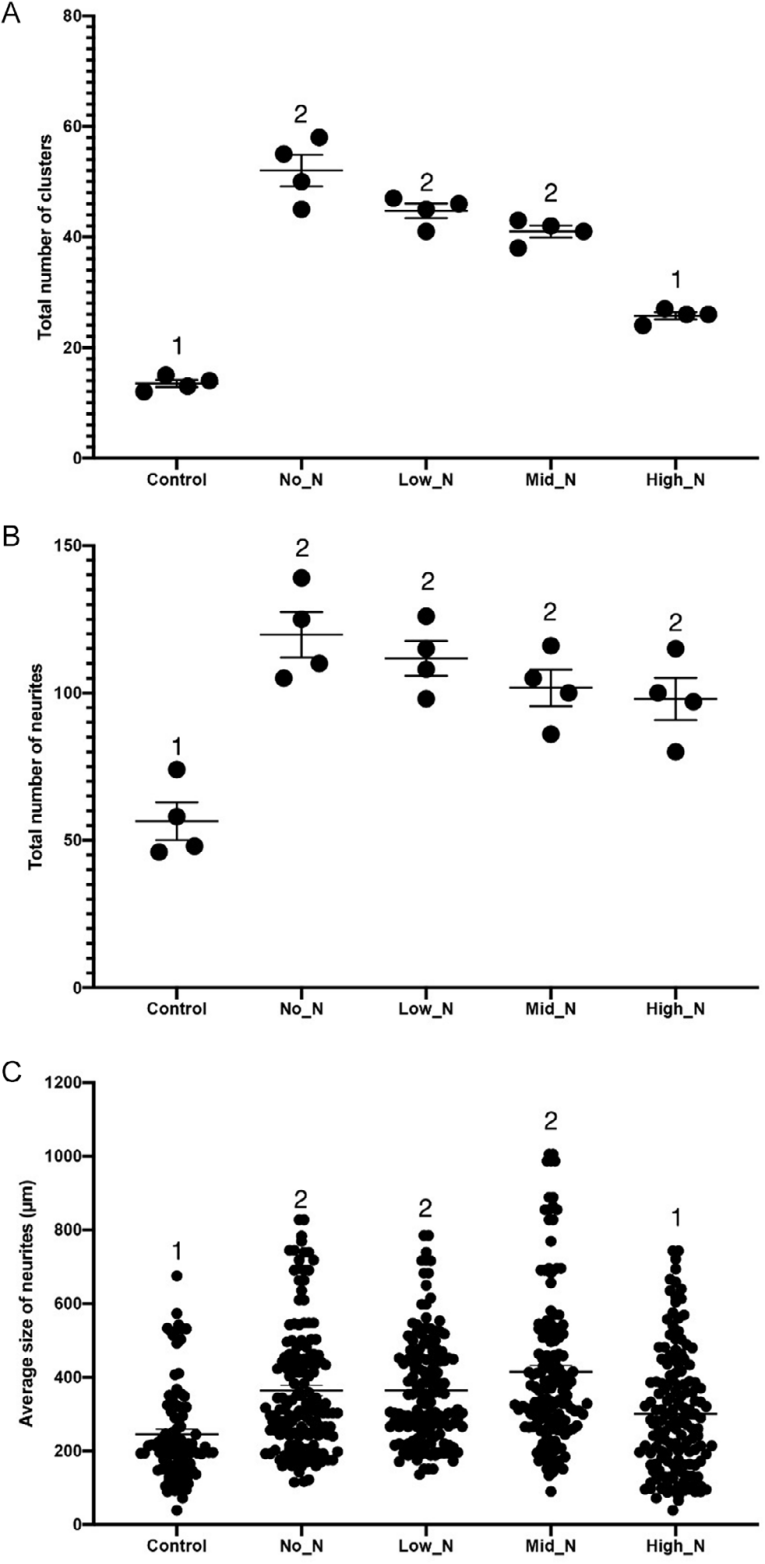
Comparison of ES differentiation on glass coverslips (control) and PACs. A) Total number of clusters. B) Total number of neurites. C) Average size of neurites. Total number of neuronal cell clusters, neurites, and neurite length were measured using ImageJ and individual values were plotted using GraphPad Prism (*n* = 4, three independent experiments). Groups not sharing a number are significantly different from each other.

Because mouse ES cells do not easily adhere to glass, we tried using PAC deposited on glass coverslips with different formulations of nitrogen and argon to promote mouse ES cell attachment and subsequent differentiation. We observed a greater attachment of cells in all conditions compared to the untreated glass coverslip (**Figures** **8** and **9**), similar to the enhancement of mouse ES cell attachment observed on plasma‐treated polycarbonate reported by Main et al.^[^ ^22^ ^]^ Furthermore, we observed greater numbers of embryoid bodies with neurite projections in all PAC conditions compared to the bare glass control (Figure 8, dotted box and arrow, respectively). We then investigated the efficiency of neuronal lineage differentiation from mouse ES cells under different compositions of nitrogen and argon.

All PACs, except for the High_N coating, led to significantly greater numbers of neuronal clusters differentiating from mouse ES cells (Figure 9a, *p* < 0.05). Neuronal clusters were defined as an attached embryoid body with neurites stemming from the periphery of the body. Additionally, the number of neurites projecting from the clusters was significantly greater in all PACs compared to the untreated control (Figure 9b, *p* < 0.05). The size of these neurites was significantly greater in all PACs except for the High_N coating (Figure 9c, *p* < 0.05). Remarkably, this neuronal cell differentiation was achieved without preattached proteins such as gelatin or Matrigel or the polysynthetic amino acid poly‐l‐lysine.

**Figure 10:**
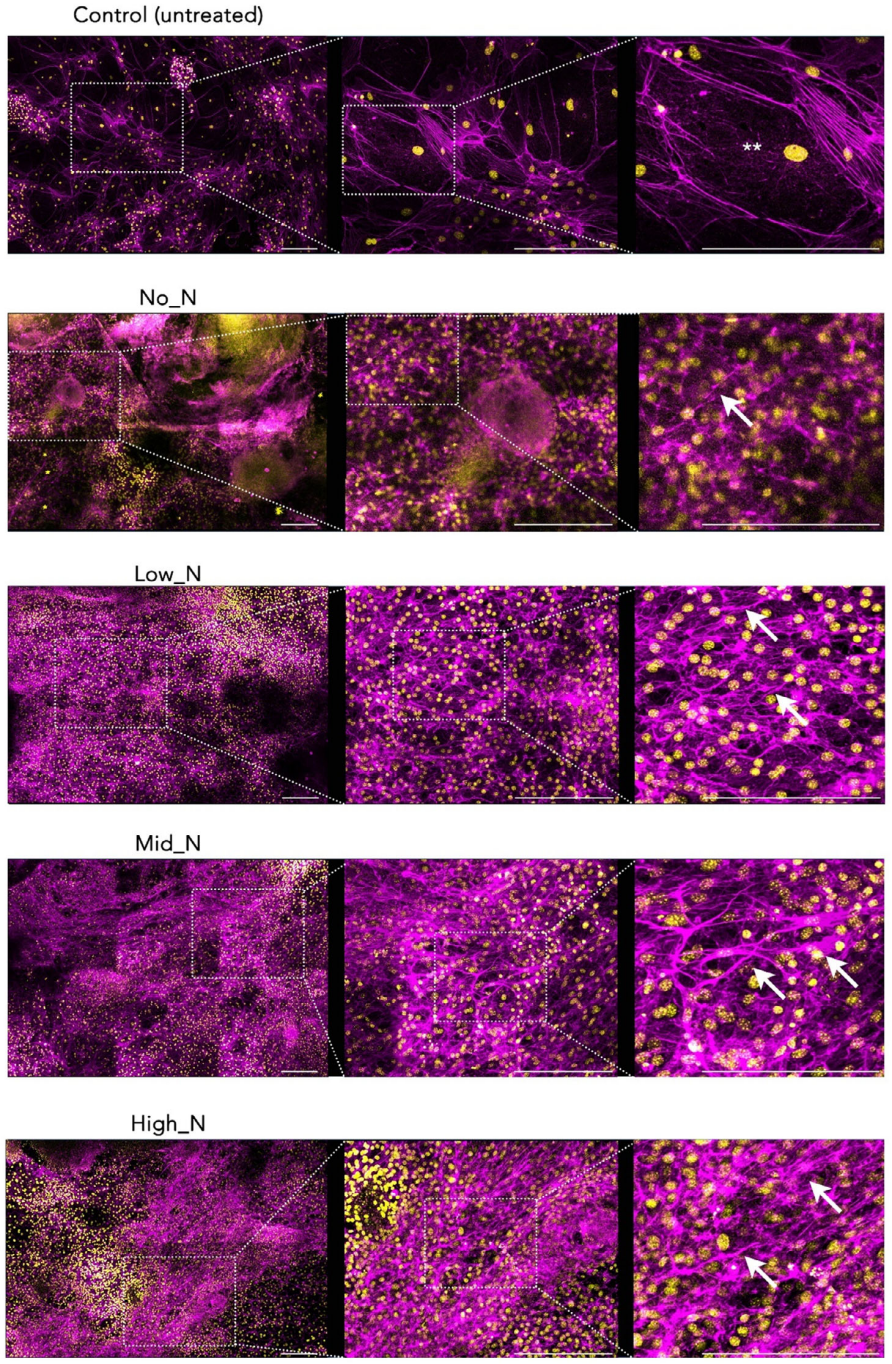
Mouse ES cells attached to PAC glass coverslips preferentially differentiate into neuronal‐like cells. Differentiated mouse ES cells were stained with F‐actin marker Alexa Fluor 555 Phalloidin (magenta) and nuclei maker DAPI (yellow) and imaged on a Leica LSM800 confocal microscope. Scale bars indicate a length of 200 μm; ** denotes surface ectoderm differentiation was found on bare glass (Control) while white arrowheads denote neural cell differentiation was dominated on PACs. Images on the same row from the left to the right show increasing magnification of the rectangular areas.

**Figure 11:**
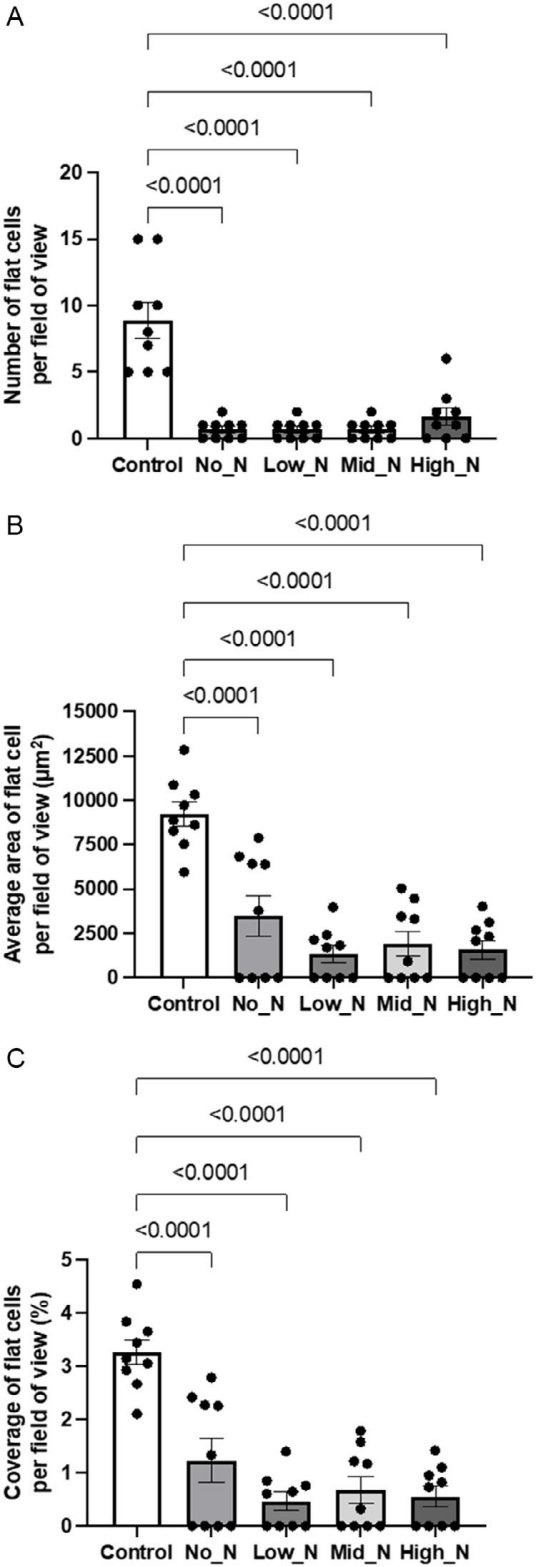
Comparison of flat cells on bare glass coverslips with PAC‐treated glass coverslips. A) Total number of flat cells counted per field of view (*n* = 9). B) The average area and C) percent coverage of flat cells per field of view (*n* = 9). Area calculated according to Phalloidin and DAPI staining using Image J and plotted on GraphPad Prism v7. Data presented as mean ± SEM. Significant differences between flat cells on untreated and PAC glass were determined by a two‐tailed, one‐way ANOVA with Tukey's post hoc analysis test. A *p‐*value less than 0.05 was deemed significant.

Confocal imaging was performed on differentiation cultures in all conditions to better understand the morphology of these neurites (**Figure** **10**). Mouse ES cell‐derived neurons were stained with Phalloidin to visualize F‐actin staining of the neurites (Figure 10, magenta). Cells attached on the control coverslip were sparse, large, and flat, resembling surface ectoderm differentiation (Figure 10, **). Neurite coverage was expansive in all formulations of PAC glass coverslips (Figure 10, arrowheads) with no discernible differences in morphology between the four different PACs by confocal microscopy. **Figure** **11** compares the total number of flat cells and their average coverage area per field of view. Bare glass samples have significantly higher number of flat cells which occupy a larger area compared to PAC‐treated coverslips.

### 3.6 Investigation of Protein Attachment to PACs from Defined Cell Culture Media Using Mass Spectroscopy

1D Liquid chromatography mass spectrometry (LCMS) was used to distinguish between absorbed and covalently attached proteins from the neural induction culture media. This analysis aimed to investigate disparities in the protein corona and assess their connection to the neural cell differentiation and surface radical density. Proteins were identified from the LCMS data using the MASCOT database, resulting in 91 unique proteins identified across all ten conditions. After filtering, a total of 22 unique proteins were characterized by their molecular weight, predicted isoelectric point, and net charge at pH 7.4. The number of unique proteins identified per sample is summarized in **Table** **3**.

**Table 3:** Total number of unique proteins identified and total peptide spectral matches detected on all surfaces with and without SDS washing

	Mild wash		SDS wash	
	Proteins	PSM	Proteins	PSM
Glass	50	901	22	1142
No nitrogen	39	309	36	1259
Low nitrogen	46	635	34	878
Mid nitrogen	12	192	11	139
High nitrogen	24	196	9	108

**Figure 12:**
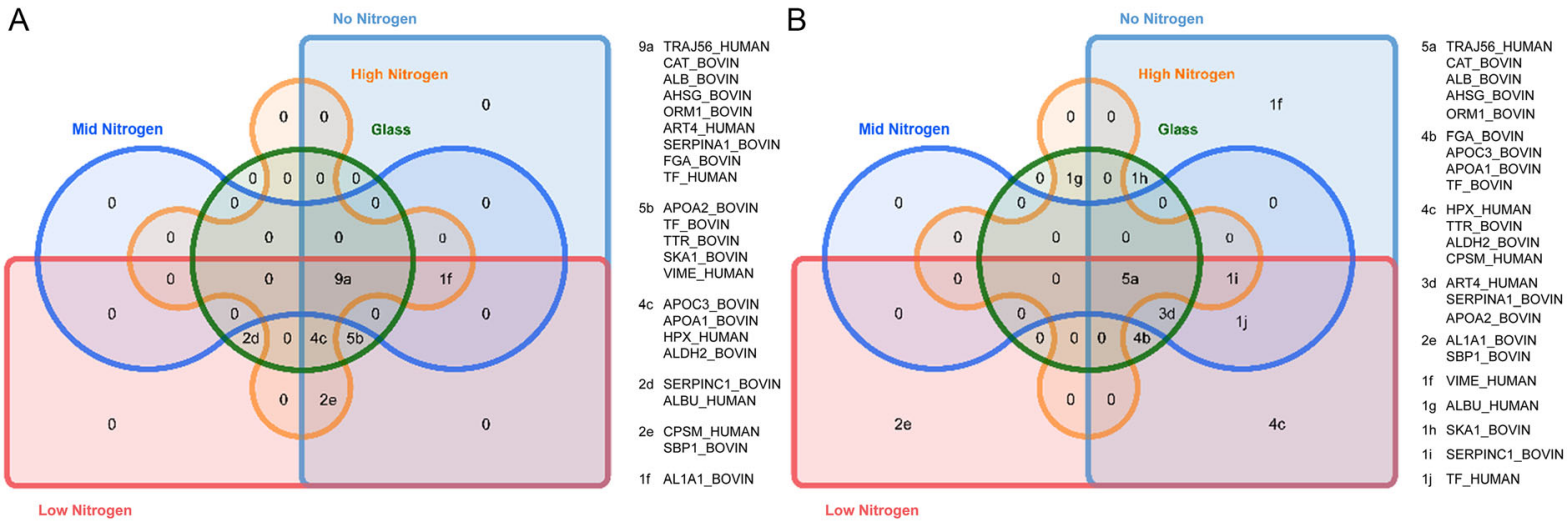
Venn diagrams comparing the number of shared versus unique proteins on untreated glass and all PAC recipes A) before and B) after washing with 5% SDS. All surfaces showed a decrease in the total number of proteins present after washing. On postwashed samples the increasing concentrations of nitrogen in the PAC correlated with a decrease in the total amount of protein detected along with fewer types of protein.

**Figure 13:**
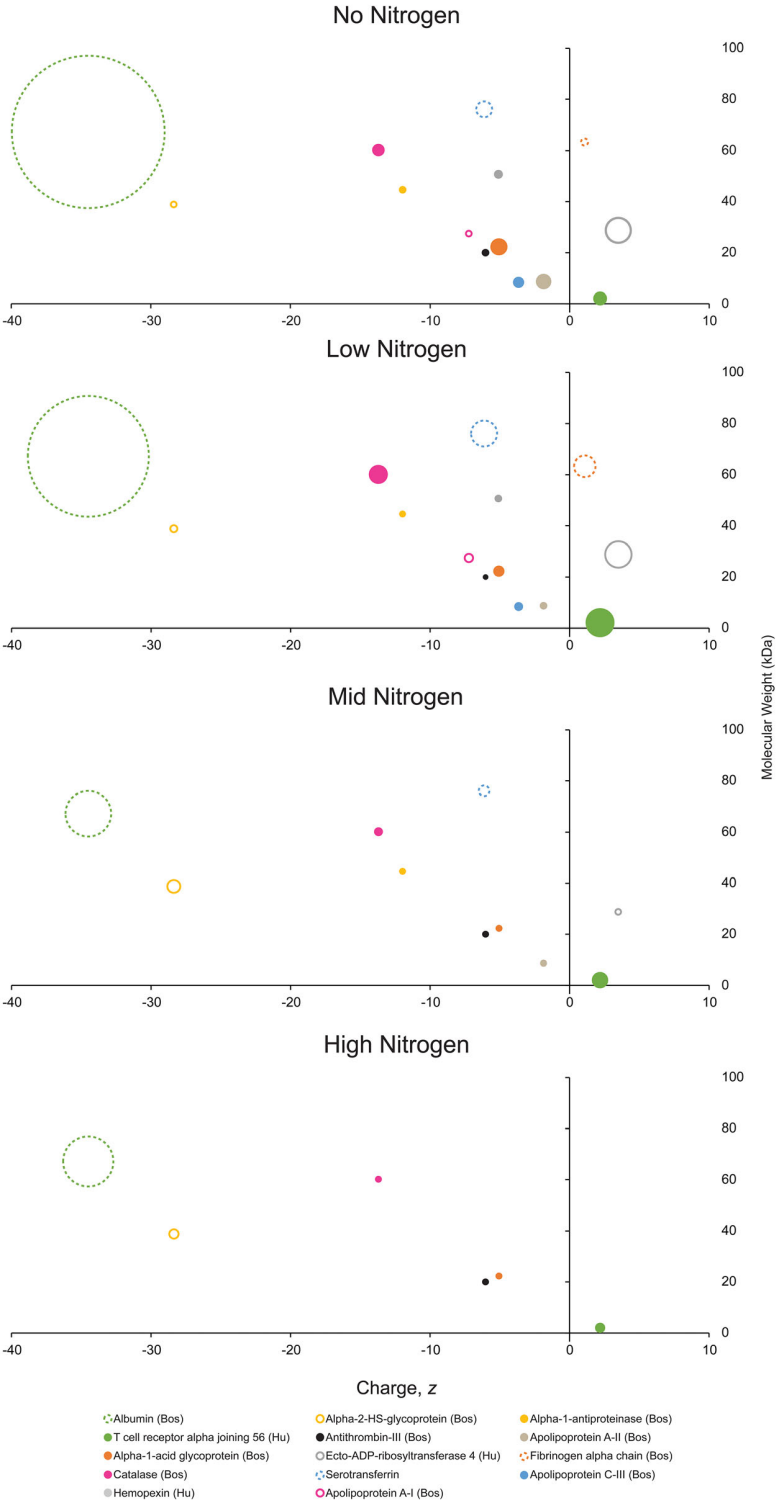
Scatter plots comparing the molecular weight (kDa) and charge (mV) of the highest ranked proteins for each PAC recipe. The size of each marker is relative to the total peptide spectral matches for each protein. Albumin and serotransferrin PSM values are the sum of both human and bovine sources due to the high degree of similarity of the proteins.

**Figure** **12** shows the overlap of the 23 identified proteins across each of the PAC types compared with untreated glass without SDS washing (A) and with SDS washing (B). Following SDS washing of the 23 proteins, 7 were unique to either or both No_N and Low_N PAC, whereas there were no unique proteins on either Mid_N or High_N. The top 14 abundant proteins are characterized in **Figure** **13** comparing the identified protein charge, molecular weight, and the total number of peptide spectral matches for each of the four PAC surfaces after washing with SDS. The nitrogen content in the PAC affected both the diversity of the protein layer and the total abundance of protein, as inferred from Table 3. Among the most abundant proteins, No_N and Low_N PACs contained more positively charged proteins, which is likely due to these surfaces having a more negative surface charge relative to Mid_N and High_N PACs. The increased abundance of positively charged proteins correlated with increased diversity of small (<40 kDa) negatively charged proteins. As the nitrogen content decreased, larger, mildly negatively charged proteins were also found in higher abundance, notably catalase and serotransferrin. Only two large proteins with a charge <−25 mV were observed on all surfaces with albumin, following the same inverse correlation with increasing nitrogen content. In contrast, alpha‐2‐HS‐glycoprotein only showed a slight but opposing trend with regard to nitrogen content. However, as all characterized proteins observed—aside from albumin, catalase, and serotransferrin—originate from the impurities in the bovine serum albumin fraction in B27 media, only general trends based on the size, charge, and molecular weight will be discussed.

## 4 Discussion

In the plasma deposition process, the application of RF power initiates the excitation and ionization of gas molecules through collisions with a few initial free electrons energized by the applied electric field. This sets off a cascade effect, generating more free electrons and positive ions. The visible glow of plasma observed through the quartz window of the plasma chamber arises from photon emission as excited species return to their ground states. The color of the glow shifts from dark pink to light pink with reduced nitrogen content in the gas mixture. Reactive species arising from acetylene and nitrogen fragments react on the substrate forming the PAC film while the positively charged species, accelerated by the −500 V bias pulses applied to the stainless‐steel sample holder, bombard the growing film, breaking bonds and creating embedded radicals. As an inert gas, argon contributes solely to the bombardment of the film by positive ions and not to its growth. Argon plasma is commonly used for cleaning organic matter residues due to its etching properties.^[^ ^23^ ^]^ Such etching by argon ions reduces the deposition rate in gas mixtures with high argon fractions. Hence, the No_N recipe employing only acetylene and argon yielded a very thin coating and this thickness remained constant even with increased deposition time. Similar observations were found with the Low_N recipe. In contrast, the Mid_N and High_N recipes produced thicker coatings and the thickness significantly increased with prolonged deposition time. The light transmittance through carbon film is significantly influenced by the film thickness.^[^ ^24^ ^]^ Despite the variations in chemistry, those PACs with thicknesses below 15 nm have no discernible impact on light transmittance. In case of high deposition rate recipes such as High_N, deposition time could be reduced to obtain thin films that maintain transparency of the glass substrates.

Due to the ion bombardment during its deposition, PAC contains embedded radicals (Figure 5). High concentrations of radicals in cell medium can be cytotoxic, inducing DNA, protein, and lipid damage, and even cell death.^[^ ^25^ ^]^ Stewart et al.^[^ ^26^ ^]^ investigated the effects of radicals embedded in plasma coatings on cells and observed no effects on either primary cells or cell lines. This can be explained by noting that the radicals embedded in coatings used for surface functionalization are not free to move outside the coating. When they reach the surface, they are often quenched through reactions with oxygen, as observed by progressive surface oxidation of the coatings. They also react with side chain groups^[^ ^27^ ^]^ of more complex molecules, such as proteins, when they are present, covalently binding them to the surface. Reactions with the protein backbone may occur at the α‐carbon sites,^[^ ^27^ ^]^ but do not propagate through the peptide bonds along the protein backbone^[^ ^28^ ^]^ and hence are not able to move into the medium or into cells to cause cytotoxicity.

The use of PAC offers several advantages over traditional methods of glass modification. First, PAC treatment enables direct covalent bonding with biomolecules due to the presence of long‐lived radicals within the coatings. This eliminates the need for multiple steps of aminosilanization or adding function groups from plasma polymerization strategies, allowing biomolecules to be immobilized onto PAC‐treated glass in a single step. The efficacy of PAC treatment in immobilization of proteins is evident from the experiment where gelatin attached strongly to PACs, exhibiting sharp printed lines (Figure 7) even after mild and stringent washes. Similarly, success has been achieved with plasma immersion ion implantation treatments of PS,^[^ ^29^ ^]^ where stable microcontact printing on radical‐rich surfaces resulted in sharp edges while the same protein patterns stamped onto untreated PS showed significant blurring of the patterns when exposed to cell culture. The improved microcontact print fidelity is promising for cell culture applications requiring small features with high resolution or close proximity such as for differentiation into multiple cell types in desired spatial configurations. The addition of PAC on glass has very minimal influence on the transparency and autofluorescence of glass substrates (Figure 1).

Second, PAC treatment offers flexibility in altering surface chemistry while maintaining long‐lived radicals. We observed that surface chemistry plays an important role in biomolecule attachment, apart from radical content. In a study by Gleize et al.^[^ ^16^ ^]^ PAC treatment on 96‐well plates was compared with plasma immersion ion implantation (PIII) treatment. PAC‐treated surfaces exhibited higher binding of DNA molecules compared to PIII‐treated surfaces, despite having lower radical content but higher nitrogen content. Another study on stainless steel demonstrated that PACs with high nitrogen content significantly improved antithrombogenicity,^[^ ^30^ ^]^ delaying thrombus formation better than those with low nitrogen or high oxygen content. By manipulating the gas composition during the plasma deposition, we demonstrated the ability to alter the elemental composition (Figure 6) and functional groups (Figure 4).

Surface chemistry, particularly surface charge, has a profound influence on biomolecule interactions and subsequent immobilized biomolecule density. In our study, we deposited four PACs with varying nitrogen content on glass coverslips to study their effect on mouse ES cell attachment and differentiation. Surprisingly, despite being nanometer‐thin, PACs had a significant impact on the attached cell density and cell fate. Mass spectrometry was used to elucidate the differences in surface‐bound protein profiles which was hypothesized to be the primary influential factor affecting cell attachment and differentiation in our experiments (Figures 12 and 13). Surface chemistry, charge, and radical density of the PAC varied with regard to nitrogen content and these properties are likely to alter protein attachment. The immobilization of proteins and other molecules from the culture media onto a glass surface occurs in two steps: first, the molecules approach the surface, driven by long‐range and short‐range forces, and then they interact with the surface, rearranging to reduce to free energy of the protein–surface complex. The variety of molecules approaching the surface is determined by the surface charge of substrates and the molecules in the culture media. Based on the profile distribution of proteins and their charges on PAC surfaces (Figure 12), we hypothesize that there was a relationship between the surface charge and protein diversity. In particular, PAC surfaces with low nitrogen content have a greater concentration of carboxylic groups relative to amine groups and hence tend to attract positively charged molecules. Small positive proteins are more dynamic and hence would have been the first to arrive at the surface followed by the progressive arrival of small negatively charged proteins and finally the large negatively charged proteins. On the contrary, PACs with high nitrogen content tend to have more positively charged groups. Very few, small, positively charged proteins were observed on the surface while mostly large and negatively charged proteins in culture media were found on the surface. Although 13 of the 22 proteins were also found in abundance on untreated glass, the biological response of mouse ES cells to these proteins was measurably different (Figure 9) to the same or similar proteins on No_N, Low_N, and Mid_N PAC. The major difference is that those proteins are physisorbed on the glass surface while they are covalently immobilized on PAC surfaces. On the glass surface, the molecules temporarily adsorb to the surface and can easily be displaced by other molecules with higher surface affinity (Vroman effect^[^ ^31^ ^]^) or by forces transmitted through focal adhesions. On PAC, radicals emerging from the coatings react with the adsorbed molecules to form covalent bonds and hence prevent the displacement. Radical density plays an important role in this second step. This is evidenced by the higher number of proteins detected on No_N and Low_N surfaces after SDS wash (Table 3) which have higher radical density than the Mid_N and High_N surfaces (Figure 5B).

Our study shows that mouse ES cells, when cultured on PAC‐treated glass, show profound changes in differentiation compared to the same cells grown on untreated glass. PAC‐treated glass induced and promoted cell attachment, survival, and neurogenesis whereas untreated glass showed reduced cell attachment and greater differentiation into keratinocytes (skin cells). Differentiation of mouse ES cells in the media used in this study leads to the generation of ectodermal progenitors which then proceed through a lineage decision‐making process into neurons (neurectoderm) or keratinocytes (surface ectoderm) depending on the signals received by the cells. Interestingly, in this study, we did not coat the coverslips intentionally with any defined biomolecules typically used to drive lineage commitment such as growth factors or ECM.^[^ ^32^ ^]^ However, we did detect serum proteins including fibrinogen A (FGA), covalently bound to the surface. Fibrinogen‐coated plates can support iPSC attachment and differentiation effectively.^[^ ^33^ ^]^ Mouse ES cells also express CD61, part of the cell surface receptor for fibrinogen (data not shown). One potential mechanism for the enhancement in neurogenesis on PAC‐treated glass coverslips is that fibrinogen—present in minuscule amounts—is being enriched and covalently bound to the surface offering an improved substrate for ES cell attachment and neural differentiation. Other serum and liquid medium‐derived molecules may also be binding covalently and assisting in ES cell attachment and differentiation.

Future investigations will focus on correlating the immobilized molecules on the PAC surfaces with the specific neuronal cell types formed, enabling the development of neural networks with predefined surface chemistry and micropatterns.

## 5 Conclusion

The deposition of PAC is a revolutionary transformation in the surface chemistry of glass, making it a game‐changer for cell culture assay. Particularly, the differentiation of mouse ES cells into neuronal‐like cells has been demonstrated to be a straightforward process on PAC‐treated glass coverslips, without the need for conventional, prefunctionalized ECM strategies. These PACs contain long‐lived radicals which irreversibly bind biomolecules from cell culture media, creating a signaling interface that directs mouse ES cell differentiation. PACs with low nitrogen content have been observed to promote the formation of denser, larger neurite clusters compared to those with high nitrogen content and bare glass. By adjusting the ratio of precursor gases introduced into plasma, the surface chemistry, transparency, and radical density of PACs can be controlled. In‐depth analysis using mass spectrometry has revealed distinct profiles of immobilized biomolecules at the cell–surface interface, providing further insight into the differences observed in mouse ES cell differentiation. The utilization of PAC‐functionalized glass coverslips holds great promise for simplifying cell culture processes, reducing complications and serving as a low‐cost and effective platform for stem cell research.

## Conflict of Interest

The authors declare no conflict of interest.

## Data Availability

The data that support the findings of this study are available from the corresponding author upon reasonable request.
